# Simultaneous Changes in Astigmatism with Noncycloplegia Refraction and Ocular Biometry in Chinese Primary Schoolchildren

**DOI:** 10.1155/2019/5613986

**Published:** 2019-06-23

**Authors:** Yaoyao Lin, Dandan Jiang, Chunchun Li, Xiao Chang, Balamurali Vasudevan, Xiaoqiong Huang, Wenzhe Zhou, Lei Qin, Yanyan Chen

**Affiliations:** ^1^School of Optometry and Ophthalmology, Wenzhou Medical University, Wenzhou, Zhejiang, China; ^2^The Eye Hospital, Wenzhou Medical University, Wenzhou, Zhejiang, China; ^3^College of Optometry, Mid Western University, Glendale, AZ, USA

## Abstract

**Purpose:**

To assess the changing profile of astigmatism in Chinese schoolchildren and the association between astigmatism changes and ocular biometry.

**Methods:**

We examined and followed up 1,463 children aged 6–9 years from Wenzhou, China. We measured noncycloplegic refraction twice each year and tested axial length (AL) and corneal radius of curvature (CRC) annually for two years. We defined clinically significant astigmatism (CSA) as ≤−0.75 diopter (D) and non-CSA astigmatism as ≤0 to >−0.75 D.

**Results:**

Prevalence of CSA at baseline was 22.4% (*n* = 327) and decreased to 20.3% (*n* = 297) at the two-year follow-up (*P* = 0.046). Ninety-two (8.1%) non-CSA children developed CSA. In multiple regression, after adjusting for age, gender, baseline cylinder refraction, and axis, children who had longer baseline ALs (>23.58 mm; odds ratio (OR) = 5.19, 95% confidence interval (CI): 2.72–9.90) and longer baseline AL/CRC ratio (>2.99, OR = 4.99, 95% CI: 2.37–10.51) were more likely to develop CSA after two years. Four-hundred and two (27.5%) children had increased astigmatism, 783 (53.5%) had decreased, and 278 (19.0%) had no change during the two-year follow-up. Children with increased astigmatism had longer baseline ALs (23.33 mm, *P* < 0.001), higher AL/CRC ratios (2.99 mm, *P* < 0.001), and more negative spherical equivalent refraction (SER) (−0.63 D, *P* < 0.001) compared with the decreased and no astigmatism change subgroups. Also, children in the increased astigmatism subgroup had more AL growth (0.68 mm, *P* < 0.001), higher increases in AL/CRC ratio (0.08, *P* < 0.001), and more negative SER change (−0.86 D, *P* < 0.001) compared with the decreased and no astigmatism change subgroups.

**Conclusions:**

The prevalence of astigmatism decreased slightly over the two-year study period. Longer ALs and higher AL/CRC ratios were independent risk factors for developing CSA. Increased astigmatism was associated with AL growth, AL/CRC ratio increases, and the development of myopia. This trial is registered with ChiCTR1800019915.

## 1. Introduction

Astigmatism is a frequent, correctable cause of visual impairment in children, whether or not this coexists with myopia or hyperopia [1]. We know that the high prevalence of astigmatism at birth decreases throughout infancy [2], but its change with age is less certain. In a longitudinal study in the USA, Harvey et al. [3] reported that schoolchildren showed clinically stable astigmatic refractions. However, in Taiwan, Chan et al. [4] found that Chinese primary schoolchildren showed a decrease in astigmatism at the one-year follow-up. Although the prevalence of astigmatism may decrease during the school years, changes in astigmatism in individual children vary.

In European children (Pärssinen et al. [5]) and native American populations (Twelker et al. [6]), the presence of astigmatism predisposes development of progressive myopia. In Twelker's et al.'s [6] study of native American population, Dobson et al. [7] found rates of myopia progression in astigmatic and nonastigmatic preschool children over a 4- to 8-year follow-up to be similar. Pärssinen [8] observed that myopia progression appeared unrelated to the initial astigmatism. Thus, the association between astigmatism and myopia is controversial [9].

Despite a large refractive database of Chinese schoolchildren, the changing profile of astigmatism has not been reported, and the relationship between the change in astigmatism and myopia is not clear in the literature. Two studies [10, 11] found axial length (AL) growth to be a more accurate predictor of myopic shift. Ratio of AL to corneal radius of curvature (CRC) (AL/CRC ratio) is an objective measure that can be used as a proxy for refractive error in the absence of cycloplegic refraction [12].

Hence, the study aims to investigate the prevalence of astigmatism, its changing profile, and how its change is associated with ocular biometry as surrogate for refractive error in 6–9-year-old Chinese schoolchildren.

## 2. Methods

### 2.1. Design and Subjects

Our study was a prospective, school-based investigation using random cluster sampling. Three schools were selected. Fifty-six children with ocular diseases or contact lens wear were excluded, and 1523 children participated. Of the enrolled children, 1463 (96.1%) completed all the eye examinations during the two-year follow-up. The purpose and details of the study examination were explained to participating parents and children before obtaining parental consent. This study was approved by the Ethics Committee of the Eye Hospital of Wenzhou Medical University and followed the tenets of the Declaration of Helsinki.

### 2.2. Procedures

Each school provided a private room where vision screenings were conducted by four professional optometrists. Before the examination, each child was informed again about the purpose and procedure of every technique. Once the children met all the requirements, examination commenced. Manifest (noncycloplegic) refraction was assessed each semester (5 times total). We used a Topcon RM8900 autorefractor (Topcon Co., Tokyo, Japan) to measure each eye at least three times to determine an average refractive error. Each eye was examined again if one value deviated from the other two by ≥±0.50 diopters (D). The IOL Master (Carl Zeiss Meditec) was used to measure AL and CRC every year.

### 2.3. Definitions

Refractive data for both eyes of each child were strongly correlated (Spearman's *ρ* 0.78–0.90, all *P* < 0.001), so only the right eye data were analyzed. Children with astigmatism ≤−0.75 D were classified as having clinically significant astigmatism (CSA), and those with astigmatism ≤0 to >−0.75 D were classified as non-CSA. The spherical equivalent of refraction (SER) was calculated as the sphere value plus half the cylinder value. Refraction was defined by spherical equivalent: myopia as ≤−0.5 D, hyperopia as ≥+0.5 D, and emmetropia as −0.5 D < SER <+0.5 D. Axis of 180° ± 15° was defined as with-the-rule (WTR), axis of 90° ± 15° as against-the-rule (ATR), and intermediate values as oblique (OBL). These standards were chosen for better comparison with other studies [4, 13–16].

### 2.4. Statistical Analysis

Statistical analysis was performed using SPSS (version 18.0). The means ± standard deviations (SD) were calculated for normally distributed data. The Pearson *χ* ^2^ test was used to compare categorical variables and *t*-tests for continuous variables. Multiple sets of continuous variables were analyzed using the ANOVA test. Multiple logistic regression was utilized to examine the effect of various factors on the dependent variable (e.g., children who developed CSA or remained as non-CSA). Two-tailed *P* values were used in all analyses, and *P* < 0.05 was considered statistically significant.

## 3. Results

### 3.1. Astigmatism Prevalence

Participants comprised 787 (53.8%) boys and 676 (46.2%) girls. The age was 7.3 ± 0.9 years (range 6 to 9 years). At baseline, the cylinder refraction for all children was −0.52 ± 0.63 D (range −5.75 D to 0 D). For children with non-CSA, the cylinder refraction was −0.27 ± 0.22 D and −1.40 ± 0.79 D for children who had CSA. The prevalence of CSA was 22.4% (*n* = 327). There was no significant difference for age (*χ* ^2^ = 3.94, *P*=0.27) or gender (*χ* ^2^ = 0.27, *P*=0.61). Of the 327 children with CSA, 249 (76.1%) had WTR astigmatism, 11 (3.4%) had ATR, and 67 (20.5%) had OBL astigmatism. The mean cylinder refraction and axis of CSA did not differ across each age group (*F* = 0.53, *P*=0.670; *χ* ^2^ = 3.81, *P*=0.700) (Table 1).

### 3.2. Changes in Astigmatism

**Figure 1:**
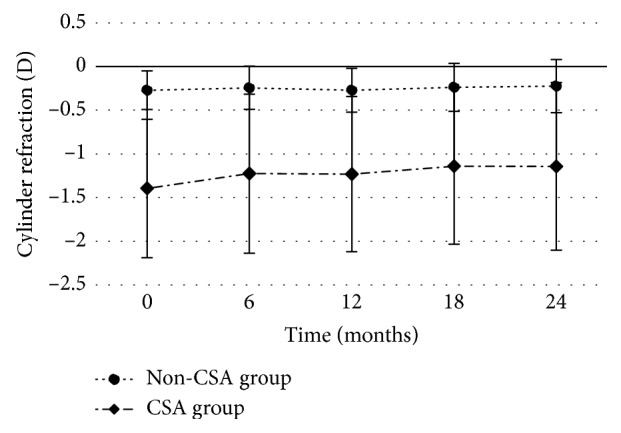
CSA and non-CSA changes in cylinder refraction.

**Figure 2:**
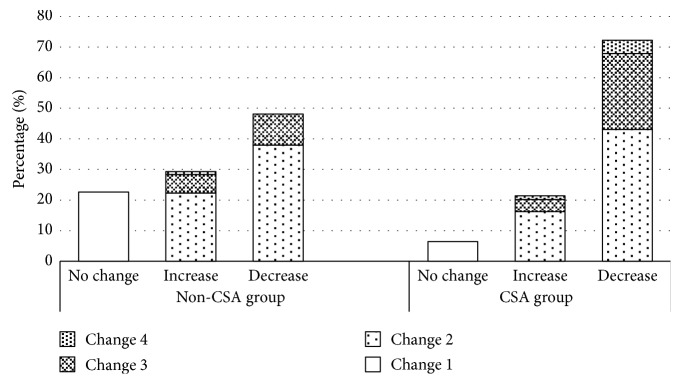
Absolute value of dioptric changes in cylinder refraction changes in the non-CSA and CSA groups. No-change subgroup, no change in diopters; increase subgroup, increases in diopters; decrease subgroup, decreases in diopters. Change 1: dioptric change = 0 D; Change 2: dioptric change >0 to <0.5 D; Change 3: dioptric change ≥0.5 D to <1.0 D; Change 4: dioptric change ≥1.0 D.

Cylinder refraction in all children changed from −0.52 ± 0.63 D to −0.43 ± 0.65 D (*P* < 0.001) after two years. In the children with CSA, cylinder refraction decreased from −1.40 ± 0.79 D to −1.14 ± 0.96 D (*P* < 0.001). In the children with non-CSA, cylinder refraction decreased from −0.27 ± 0.22 D to −0.22 ± 0.30 D (*P* < 0.001, Figure 1). The prevalence of CSA decreased from 22.4% to 20.3% (*n* = 297) by study completion (*χ* ^2^ = 467.72, *P* < 0.001). In the non-CSA group (*n* = 1,136), astigmatism increased for 29.2% (*n* = 332) of the children, decreased for 48.2% (*n* = 547), and did not change for 22.6% (*n* = 257). In the CSA group (*n* = 327), astigmatism increased for 21.4% (*n* = 70) of the children, decreased for 72.2% (*n* = 236), and did not change for 6.4% (*n* = 21). Most of the absolute dioptric changes in cylinder refraction were between >0 and < 0.5 D for the two groups (Figure 2). Such changes occurred in 59.3% (*n* = 194) of the CSA children and 60.2% (*n* = 684) of the non-CSA children. In another, such changes occurred in 76.1% (*n* = 306) of the increased subgroup. Change of ≥0.5 D to <1.0 D occurred in 28.8% (*n* = 94) of the CSA children and 16.2% (*n* = 184) of the non-CSA children. Also, such changes occurred in 20.1% (*n* = 81) of the increased subgroup. Change of ≥1.0 D occurred in 5.5% (*n* = 18) of the CSA children and 1.0% (*n* = 11) of the non-CSA children. And, such changes occurred in 3.7% (*n* = 15) of the increased subgroup.  Table 2 showed the proportion of the type of astigmatism changes. There was a significant difference between the baseline and final examination for the proportion of the type of astigmatism (*χ* ^2^ = 71.66, *P* < 0.001). The proportion of CSA children who had hyperopic astigmatism decreased from 22.0% (72/327) to 12.5% (37/297), and the proportion with mixed astigmatism decreased from 43.4% to 26.9%. However, the proportion with myopic astigmatism increased from 34.6% to 60.6% at the two-year follow-up. For the astigmatism increased subgroup, hyperopic astigmatism decreased from 2.0% (8/402) to 1.7% (7/402), mixed astigmatism increased from 5.5% (22/402) to 9.2% (37/402), and myopic astigmatism increased from 10.0% (40/402) to 29.4% (118/402) (*χ* ^2^ = 100.57, *P* < 0.001).

### 3.3. Association between Change of Astigmatism and Ocular Biometry

For non-CSA children, 8.1% (*n* = 92) developed CSA and 91.9% (*n* = 1,044) remained non-CSA. In the multiple logistic regression model (Table 3), after adjusting for age, gender, baseline cylinder refraction, and baseline axis of astigmatism, the higher baseline AL (odds ratio [OR] = 5.19, 95% confidence interval [CI]: 2.72–9.90 for the top quartile compared with the bottom quartile) was significantly associated with the development of CSA from non-CSA eyes. Similarly, the higher AL/CRC ratio (OR = 4.99, 95% CI: 2.37–10.51 for the top quartile compared with the bottom quartile) was also significantly associated with the development of CSA from non-CSA eyes. However, there were no differences between the ALs of 22.53 to 23.58 mm and the ALs <22.53 mm for the development of CSA from non-CSA. AL/CRC ratios of 2.89 to 2.99 were also not associated with the development of CSA compared with the bottom quartile. In another, 73.9% (68/92) developed myopic astigmatism of children who had non-CSA at baseline. Of them, the radius of CR of the horizontal meridian increased from 7.90 mm to 7.94 mm after the two-year follow-up (*P* = 0.02). However, there was no significant difference for the change of the radius of CR of the steep meridian (*P* = 0.84).

The percentage of baseline ALs (>23.58 mm) in the top quartile of non-CSA eyes was significantly higher in myopes (47.8%) compared with emmetropes (19.2%) and hyperopes (7.1%) (*P* < 0.001 each, Table 4). However, the percentage of baseline ALs (<22.53 mm) in the bottom quartile of non-CSA eyes was significantly higher in hyperopes (44.0%) compared with emmetropes (25.4%) and myopes (12.4%) (*P* < 0.001 each). For baseline AL/CRC ratios (>2.99), the percentage of eyes in the top quartile was higher in myopes (52.5%) compared with emmetropes (15.6%) and hyperopes (5.5%) (*P* < 0.001 each). However, the percentage of baseline AL/CRC ratios (<2.89) in the bottom quartile was higher in hyperopes (37.9%) compared with emmetropes (20.5%) and myopes (10.0%) (*P* < 0.001 each). The AL was 23.59 ± 0.96 mm for myopes, and it decreased to 22.97 ± 0.66 mm for emmetropes and 22.63 ± 0.76 mm for hyperopes (*F* = 103.45, *P* < 0.001). The AL/CRC ratio was 3.01 ± 0.11 for myopes, and it decreased to 2.94 ± 0.06 for emmetropes and 2.90 ± 0.08 for hyperopes (*F* = 145.16, *P* < 0.001).

Children with CSA (*n* = 327) had two outcomes after the two-year study. Astigmatism either decreased to non-CSA (37.0%, *n* = 122), or it remained CSA (63.0%, *n* = 205). After adjusting for age, gender, and baseline axis of astigmatism, the AL/CRC ratio (OR = 0.31, 95% CI: 0.15–0.64 for the top quartile compared with the bottom quartile) was associated with the decrease of CSA to non-CSA. However, the baseline AL was not associated with the decrease of CSA to non-CSA (Supplementary Table 1).

Among the study participants, 402 (27.5%) had increased astigmatism, 783 (53.5%) had decreased astigmatism, and 278 (19.0%) children had no change in astigmatism at follow-up. Using the least significant difference (LSD) pairwise comparison methods (Table 5), we found that the subgroup of children with increased CSA had longer ALs (23.33 mm), larger AL/CRC ratios (2.99), and more myopic SERs (−0.63 D) compared with children who had decreases in these biometric parameters (AL = 22.89 mm, AL/CRC ratio = 2.94, SER = −0.07 D, *P* < 0.001 for each). Similarly, the subgroup with increased CSA had longer ALs, larger AL/CRC ratios, and more myopic SERs than the subgroup that had no changes in CSA (AL = 23.06 mm, AL/CRC ratio = 2.93, SER = −0.01 D, *P* < 0.001 for each). Moreover, AL growth (0.68 mm), AL/CRC ratio change (0.08), and myopic progression (−0.86 D) were all greater in the subgroup with increased CSA compared with the subgroup with decreased CSA (AL = 0.56 mm, AL/CRC ratio = 0.07, SER = −0.31 D, *P* < 0.001 for each) and with the subgroup without change in CSA (AL = 0.53 mm, AL/CRC ratio = 0.07, SER = −0.39 D, *P* < 0.001 for each).

## 4. Discussion

### 4.1. Prevalence of Astigmatism

The prevalence of astigmatism varies according to ethnicity, population, and measurement standards. We found the prevalence of CSA at baseline (≤0.75 D, 22.4%) to be higher than findings in South African populations [17] (≤−0.75 D, 5–15 years, 9.2%) and in other populations including those in Iran (≤−0.75 D, 6–17 years, 11.5%) [18] and Nepal (≤−0.75 D, 5–15 years, 3.5%) [19]. Our prevalence was lower than that in another Chinese study [20], where prevalence was 42.7% in urban districts (≤−0.75 D, 5–15 years) and 25.3% (≤−0.75 D, 13–17 years) in rural districts [21]. Chan et al. [4] reported that 32.9% of Taiwanese schoolchildren had astigmatism >1.0 D, a prevalence higher than that of our study. We did not detect correlations with either gender or age like those reported by Chebil et al. [22] and Fotouhi et al. [9]. Our results also agreed with others [4, 23, 24], where most schoolchildren had WTR astigmatism. We found no significant association between age and CSA, consistent with data from Fotouhi et al. [9] and Chan et al. [4].

### 4.2. Changes in Astigmatism

Over the two years of this study, the prevalence of astigmatism decreased, declining in both the non-CSA group (−0.27 D to −0.22 D) and the CSA group (−1.40 D to −1.14 D). In a study of 4,662 Chinese schoolchildren (5–13 years), the magnitude of astigmatic error showed little change (0.004 D) over the 28.5-month duration follow-up [25]. Chan et al. [4] found that the cylinder refraction decreased from −0.74 D to −0.58 D after a one-year follow-up in children aged 7–11 years. However, the Northern Ireland Childhood Errors of Refraction (NICER) study [26] reported that the prevalence of 6–7 years old astigmates remained stable after a 3-year follow-up. The reasons for these differences may be attributed to the different populations and the standards used for astigmatism. Although both groups had overall reductions in astigmatism, astigmatism in diopters may increase, decrease, or remain unchanged for individual children. Dioptric changes of the absolute value of astigmatism was mostly in the range of >0 to <0.5 D in the two groups, which means that most of the changes were relatively small. In our study, we found that hyperopic astigmatism decreased and myopic astigmatism increased after two-year follow-up which was consistent with the data from Dobson et al. [7].

### 4.3. Association between Change of Astigmatism and Ocular Biometry

In our non-CSA group, 8.1% of the children developed CSA after two years. This incidence of CSA conversion from non-CSA was relatively low compared with the 11.5% of Singaporean children aged 7–9 who developed CSA (defined as cylinder refraction ≤−1.0 D) over a three-year duration [27] and the 9.1% of the children aged 6-7 years in the three years of the NICER study [26]. After accounting for the baseline age, gender, cylinder refraction, and axis of astigmatism, our multiple analyses showed that children with a baseline AL >23.58 mm, i.e., higher than the 75th percentile, were 5.19 times more likely to develop CSA. The baseline AL/CRC ratio >2.99, i.e., higher than the 75th percentile, was the independent factor most strongly associated with non-CSA developing to CSA after two years.

AL is correlated with SER in longer eyes more likely to be myopic [28]. Zhang et al. [29] reported that AL predicts the onset of myopia, and the AL/CRC ratio is strongly correlated with the SER [13, 30, 31]. AL/CRC ratio can be a useful marker of the onset and the progression of myopia [32]. Several studies [33–35] reported no significant change in the AL or AL/CRC ratio before and after mydriasis which compares well with measurements in other studies with or without cycloplegia. We speculate that eyes with ALs longer than 23.58 mm and with AL/CRC ratios higher than 2.99, both of which indicate a high likelihood of myopia, are more likely to develop CSA. In our study, the percentage of baseline AL (>23.58 mm) and AL/CRC ratio (>2.99) for the top quartile was significantly higher in myopes compared with that in emmetropes and hyperopes. The mean ALs and AL/CRC ratios were also larger among myopes than emmetropes and hyperopes, a finding consistent with our hypothesis. In a cross-sectional study, Huang et al. [36] found that myopia was associated with an increased risk of astigmatism. Tong et al. [27] reported a similar result that children who were myopic at baseline had a higher incidence of astigmatism than nonmyopes. Increased myopia is often accompanied by changes in axial length and corneal curvature [37]. In this study, we found that for non-CSA children who developed myopic astigmatism, the radius of CR of the horizontal meridian increased and the radius of CR of the steep meridian was of no change. The AL growth may cause corneal morphologic changes which result in curvature and axial asymmetries and increased chance of developing astigmatism. However, due to the limited sample size, the specific change of the radius of CR and the reasons should be further studied.

In another, among the non-CSA eyes that converted to CSA, the percentages of baseline ALs <22.53 mm and AL/CRC ratios <2.89 for the bottom quartile were significantly higher in hyperopes compared to emmetropes and myopes. The lower ALs and lower AL/CRC ratios are more likely to be in hyperopic eyes [28]. Compared with the bottom quartile for AL or AL/CRC ratio, ALs of 22.53–23.58 mm or AL/CRC ratios of 2.89–2.99 were not independent factors associated with non-CSA developing to CSA even though it has been reported that hyperopic eyes are more likely to be astigmatic than myopic eyes [7]. Fotouhi et al. [9] found that the association between astigmatism and myopia (odds ratio = 8.81) was stronger than its association with hyperopia (Odds ratio = 3.81). However, our study showed the opposite results. The relationship between astigmatism and hyperopia is still unclear and should be further studied.

Our results showed that compared with the decreased CSA subgroup and the unchanged subgroup, children in the increased CSA subgroup had longer ALs, higher AL/CRC ratios, and greater increases in AL and AL/CRC. Also, the increased astigmatism was correlated with higher myopic refraction and myopic development. This indicates that increased astigmatism is associated with visual blurring perturbations that might influence the development of myopia [38].

A limiting factor in our study was our use of manifest (noncycloplegic) refraction data. Zhang et al. [39] and Fotouhi et al. [9] both reported that dioptric astigmatism measured in children who consented to cycloplegia was similar to that measured in those who refused consent (*P*=0.248; *P*=0.296). However, obtaining refractive error in the absence of cycloplegia may overestimate myopic power and underestimate hyperopia [40]. Therefore, we used AL and AL/CRC ratio to serve as objective indicators for the development of myopia.

Other factors associated with astigmatism, such as ethnicity, body mass index, and parental astigmatism, were not included in our study. Neither did we analyze how internal astigmatism and corneal astigmatism changed, or how their individual effects related to the change of cylinder refraction.

## 5. Conclusion

The prevalence of astigmatism decreased slightly during the two-year follow-up. Children who had longer ALs and higher AL/CRC ratios were more likely to develop CSA. Increased astigmatism was associated with AL growth, AL/CRC ratio increase, and myopic development.

## Figures and Tables

**Table 1:** Cylinder refraction and axis of CSA children in different age groups.

Age (y)	Cylinder refraction^a^ (D)	*P* value^*∗*^	Axis of astigmatism^b^ (≤−0.75 D)			*P* value^#^
			WTR	ATR	OBL	
6	−1.38 ± 0.85	0.67	66 (78.6%)	2 (2.4%)	16 (19.0%)	0.7
7	−1.34 ± 0.78		88 (72.7%)	6 (5.0%)	27 (22.3%)	
8	−1.47 ± 0.78		79 (79.0%)	3 (3.0%)	18 (18.0%)	
9	−1.38 ± 0.71		16 (72.7%)	0 (0.0%)	6 (27.3%)	

D, diopters; y, years; WTR, with-the-rule; ATR, against-the-rule; OBL, oblique. ^a^Means ± standard deviations; ^b^number of eyes (%); ^*∗*^ANOVA; ^#^ *χ* ^2^ test.

**Table 2:** Comparison of the type of astigmatism at initial examination and final examination in the 1463 children who underwent follow-up examination at 2 years.

Group at baseline	Group at final (2 years later)									
	Non-CSA		Hyperopic astigmates		Mixed astigmates		Myopic astigmates		Total	
	*n*	%	*n*	%	*n*	%	*n*	%	*n*	%
Non-CSA	1044	91.9	3	0.3	21	1.8	68	6.0	1136	100
Hyperopic astigmates	34	47.2	28	38.9	6	8.3	4	5.6	72	100
Mixed astigmates	50	35.2	6	4.2	44	31	42	29.6	142	100
Myopic astigmates	38	33.6	0	0	9	8	66	58.4	113	100
Total	1166	79.7	37	2.5	80	5.5	180	12.3	1463	100

**Table 3:** Logistic regressions of baseline factors for development of CSA from non-CSA eyes after two years.

Baseline characteristic^a^	Univariate regression			Multiple regression^b^			Multiple regression^c^		
	Odds ratio	95% CI	*P* value	Odds ratio	95% CI	*P* value	Odds ratio	95% CI	*P* value
Age (y)^*∗*#^	1.32	1.05–1.67	**0.02**						

Gender (%)^*∗*#^									
Boys	Reference								
Girls	0.76	0.49–1.17	0.2						

Cylinder refraction (D)									
0 (75th percentile)	Reference			Reference			—		
−0.5 to 0	2.22	1.18–4.19	**0.014**	2.45	1.29–4.68	**0.006**	—	—	—
<−0.5 (25th percentile)	5.84	2.76–12.34	**<0.001**	8.17	3.74–17.85	**<0.001**	—	—	—

Axis (%)									
OBL	Reference			—			Reference		
Nil	0.5	0.25–0.97	**0.04**	—	—	—	0.48	0.24–0.95	**0.035**
WTR	2.01	1.24–3.24	**0.004**	—	—	—	1.98	1.21–3.22	**0.006**
ATR	0.41	0.12–1.36	0.15	—	—	—	0.43	0.13–1.46	0.18

AL (mm)									
<22.53 (25th percentile)	Reference			Reference			—		
22.53–23.58	1.07	0.56–2.05	0.844	1.26	0.65–2.46	0.49	—	—	—
>23.58 (75th pencentile)	3.96	2.13–7.36	**<0.001**	5.19	2.72–9.90	**<0.001**	—	—	—

AL/CRC ratio									
<2.89 (25th percentile)	Reference			—			Reference		
2.89–2.99	1.55	0.74–3.26	0.25	—	—	—	1.5	0.71–3.17	0.29
>2.99 (75th pencentile)	5.13	2.45–10.74	**<0.001**	—	—	—	4.99	2.37–10.51	**<0.001**

CSA, clinically significant astigmatism; 95% CI, 95% confidence interval; y, years; AL, axial length; CRC, corneal radius of curvature; D, diopters; Nil, cylinder refraction of zero. ^a^Percentiles correspond to baseline values for children with non-CSA; ^b^Logistic functions were adjusted for age, gender, and baseline cylinder refraction; ^c^Logistic function were adjusted for age, gender, and axis of baseline non-CSA (≤0 to > −0.75 D). ^*∗*^ *P* > 0.05 in multiple regression^b^; ^#^ *P* > 0.05 in multiple regression^c^.

**Table 4:** Baseline ocular biometry percentages associated with refractive status of non-CSA children.

Variables	Hyperopes (≥+0.5 D)		Emmetropes (−0.5 D to +0.5 D)		Myopes (≤−0.5 D)		*P* value^a^
	*n*	%	*n*	%	*n*	%	
AL (mm)							
<22.53 (25th percentile)	80	44	166	25.4	37	12.4	<0.001
22.53–23.58	89	48.9	362	55.4	119	39.8	
>23.58 (75th percentile)	13	7.1	125	19.2	143	47.8	

AL/CRC ratio							
<2.89 (25th percentile)	69	37.9	134	20.5	30	10	<0.001
2.89–2.99	103	56.6	417	63.9	112	37.5	
>2.99 (75th percentile)	10	5.5	102	15.6	157	52.5	

Total	182	100	653	100	299	100

AL, axial length; CRC, corneal radius of curvature; D, diopters. ^a^Determined using Pearson *χ* ^2^ test.

**Table 5:** Comparison of ocular biometry among the three astigmatism change subgroups.

Ocular parameter	Astigmatism subgroups			*F*	*P* value
	Increase	Decrease	No change		
Baseline AL (mm)	23.33 ± 0.98^*∗*#^	22.89 ± 0.84	23.06 ± 0.75	74.562	<0.001
AL change (mm)	0.68 ± 0.41^*∗*#^	0.56 ± 0.36	0.53 ± 0.33	16.466	<0.001
Baseline AL/CRC ratio	2.99 ± 0.08^*∗*#^	2.94 ± 0.08	2.93 ± 0.08	45.005	<0.001
AL/CRC ratio change	0.08 ± 0.06^*∗*#^	0.07 ± 0.04	0.07 ± 0.04	9.312	<0.001
Baseline SER (D)	−0.63 ± 1.40^*∗*#^	−0.07 ± 1.04	−0.01 ± 0.74	39.142	<0.001
SER change (D)	−0.86 ± 1.15^*∗*#^	−0.31 ± 0.86	−0.39 ± 0.77	33.222	<0.001

AL, axial length; CRC, corneal radius of curvature; SER, spherical equivalent refraction; D, diopters; values are means ± standard deviations. ^*∗*^Compared to the decrease subgroup, *P* < 0.001; ^#^compared to the no-change group, *P* < 0.001.

## Data Availability

The original data, figures, and tables that were used to support the findings of this study are available from the corresponding author upon request.

## Supplementary Materials

Supplementary Materials Supplementary Table 1 analyzed the baseline factors associated with CSA decreased to non-CSA after two years. However, the result was just the opposite of Table 2, and no more meaningful conclusions were reached.
