# Supplementary material for: Simultaneous Changes in Astigmatism with Noncycloplegia Refraction and Ocular Biometry in Chinese Primary Schoolchildren
Source: J Ophthalmol. 2019 Jun 23;2019:5613986. doi: 10.1155/2019/5613986 (PMC6612406; doi:10.1155/2019/5613986)
Supplement: Supplementary Materials — Supplementary Table 1 analyzed the baseline factors associated with CSA decreased to non-CSA after two years. However, the result was just the opposite of Table 2, and no more meaningful conclusions were reached. [file 5613986.f1.doc]

| Supplemental Table 1: Logistic regressions for baseline factors associated with CSA decreased to non-CSA after two years. | | | | | | | |
| --- | --- | --- | --- | --- | --- | --- | --- |
| Baseline Characteristic^a^ | Univariate regression | | |  | Multiple regression^b^ | | |
|  |  |  |  |  |  |  |  |
|  | Odds ratio | 95% CI | P-value |  | Odds ratio | 95% CI | P-value |
| Age (yrs)^*^ | 1.14 | 0.70-1.85 | 0.6 |  |  |  |  |
| Gender (%)^*^ |  |  |  |  |  |  |  |
| Boys | Reference |  |  |  |  |  |  |
| Girls | 0.99 | 0.53-1.83 | 0.96 |  |  |  |  |
| Axis (%) |  |  |  |  |  |  |  |
| WTR | Reference |  |  |  | Reference |  |  |
| ATR | 2.54 | 0.19-0.83 | 0.13 |  | 2.77 | 0.79-9.71 | 0.11 |
| OBL | 2.45 | 0.15-3.81 | **0.001** |  | 2.51 | 1.43-4.42 | **0.001** |
| AL(mm) |  |  |  |  |  |  |  |
| <22.23 (25th Percentile) | Reference |  |  |  | - |  |  |
| 22.23-23.57 | 0.58 | 0.34-1.00 | 0.05 |  | - | - | - |
| >23.57 (75th Pencentile) | 0.60 | 0.32-1.13 | 0.11 |  | - | - | - |
| AL/CRC ratio ratio |  |  |  |  |  |  |  |
| <2.9 (25th Percentile) | Reference |  |  |  | Reference |  |  |
| 2.9-3.01 | 0.96 | 0.56-1.63 | 0.96 |  | 0.70 | 0.52-1.55 | 0.90 |
| >3.01 (75th Pencentile) | 0.33 | 0.16-0.67 | **0.002** |  | 0.31 | 0.15-0.64 | **0.002** |
| CSA, clinically significant astigmatism; 95% CI, The 95% confidence intervals; AL, axial length; CRC, corneal radius of curvature; D, diopters; ^a^ Percentiles correspond to baseline examination values for children who had baseline CSA; ^b^ Logistic function were adjusted age, gender and baseline axis of CSA (≤-0.75D); ^*^ p>0.05 in Multiple regression^b^ . | | | | | | | |
